# CD248 expression in human adipose tissue is upregulated in obesity and following glucocorticoid exposure: a mediator of insulin resistance and adipose dysfunction?

**DOI:** 10.1186/s10020-026-01661-8

**Published:** 2026-10-07

**Authors:** Rutger Laterveer, Martin H. Lundqvist, Edward M. Conway, Afshan N. Malik, Maria K. Svensson, Magnus Sundbom, Petros Katsogiannos, Jan W. Eriksson, Maria J. Pereira

**Affiliations:** 1 Department of Medical Sciences, Clinical Diabetology and Metabolism, Uppsala University, Uppsala, 751 85 Sweden; 2 Centre for Blood Research, Life Sciences Institute, Faculty of Medicine, University of British Columbia, Vancouver, Canada; 3 Departments of Medicine and Pathology and Laboratory Medicine, Life Sciences Institute, Faculty of Medicine, University of British Columbia, Vancouver, Canada; 4 Diabetes & Obesity, School of Cardiovascular Medicine and Metabolic Sciences, King’s College London, London, UK; 5 Department of Medical Sciences, Renal Medicine, Uppsala University, Uppsala, Sweden; 6 Uppsala Clinical Research Center, Uppsala University, Uppsala, Sweden; 7 Department of Surgical Sciences, Uppsala University, Uppsala, Sweden

**Keywords:** CD248, Endosialin, Adipose tissue, Obesity, Glucocorticoids, Insulin resistance

## Abstract

**Background:**

CD248 is a transmembrane glycoprotein implicated in fibrosis, hypoxia, and insulin resistance, but its role in human adipose tissue remains incompletely understood. Because glucocorticoids regulate these processes, we investigated whether CD248 is glucocorticoid responsive and characterized its expression across adipose depots and sexes in obesity.

**Methods:**

CD248 expression was assessed in adipose tissue obtained by needle biopsy (75 male/114 female, BMI: 20.1–47.9 kg/m^2^) and surgical sampling (24 male/39 female, BMI: 20.0–54.4 kg/m^2^) from individuals without type 2 diabetes, and in an additional cohort with type 2 diabetes (21 male/33 female, BMI: 22.4–42.4 kg/m^2^). Adipose tissue, isolated adipocytes and stromal vascular fraction were exposed to cortisol and dexamethasone. Circulating CD248 was analyzed in 44,545 participants from the UK Biobank Pharma Proteomics Project.

**Results:**

CD248 expression was higher in subcutaneous adipose tissue in obesity at mRNA (86%) and protein (2-fold) levels, with BMI as the strongest independent predictor. Expression did not differ between individuals with and without type 2 diabetes. Depot-specific differences were identified, with lower CD248 expression in omental adipose tissue than subcutaneous adipose tissue and opposing BMI associations. Sex-specific differences were observed at the protein level, with higher CD248 abundance in females with obesity. Transcriptomic analysis identified remodelling-related pathways in subcutaneous adipose tissue and metabolic pathways in omental adipose tissue. Dexamethasone and cortisol increased *CD248* gene expression, and dexamethasone increased CD248 unglycosylated protein in adipocytes. Circulating CD248 increased with BMI and was higher in females with obesity, mirroring adipose tissue protein.

**Conclusion:**

Our findings identify CD248 as a glucocorticoid-responsive and depot-specific marker associated with adiposity and metabolic disturbances, suggesting a potential role in obesity-related adipose tissue dysfunction.

**Supplementary Information:**

The online version contains supplementary material available at https://doi.org/10.1186/s10020-026-01661-8.

## Introduction

Obesity is a major global health challenge, strongly linked to cardiovascular disease and type 2 diabetes (T2D). Adipose tissue plays a central role in metabolic regulation, functioning not only as an energy store but also as an endocrine organ that communicates with other tissues (Mazza et al. 2024). In obesity, white adipose tissue undergoes extensive structural and functional remodeling, including altered vascularization, inflammation, and changes in local oxygen availability. These effects can exacerbate hypoxia, fibrosis, and insulin resistance (Reyes-Farias et al. 2021). Thus, both systemic hormonal regulation and the local tissue microenvironment are central to metabolic dysfunction; yet, how these processes integrate at the cellular and molecular level remains incompletely understood.

Glucocorticoids are central to adipose function, regulating glucose and lipid metabolism, adipocyte differentiation, and stress responses (Kuckuck et al. 2024, Vali et al. 2024). Glucocorticoids are important regulators of many of these remodeling processes (Vali et al. 2024). Disruptions in glucocorticoid signaling or circadian cortisol rhythms may contribute to weight gain, visceral fat accumulation, and insulin resistance (Boyar et al. 1979, Rodrigues et al. 2024).

CD248 is a type I transmembrane glycoprotein that has been extensively studied in cancer and fibrotic disease (Li et al. 2025, Lu et al. 2024). It is predominantly expressed in stromal cell populations such as pericytes and fibroblasts, where it contributes to tissue remodeling and is upregulated under hypoxic conditions (Bartis et al. 2016, Kontsekova et al. 2016, Ohradanova et al. 2008). Although CD248 has been less studied in adipose tissue, emerging evidence suggests a role in metabolic regulation. Petrus et al. showed that CD248 expression in human adipose tissue is higher in the stromal vascular fraction (SVF) than in mature adipocytes, but only the adipocyte fraction shows a significant positive correlation with BMI (Petrus et al. 2019). CD248 expression was also increased in white adipose tissue from insulin-resistant individuals with obesity, while lower CD248 expression in human adipocytes was associated with greater insulin sensitivity (Petrus et al. 2019, Benedet et al. 2023). Furthermore, Benedet et al. demonstrated that CD248 can impair insulin signaling by binding to the insulin receptor and limiting its activation by insulin (Benedet et al. 2023). Together, these findings place CD248 at the intersection of adipose tissue remodeling, hypoxia-related pathways, and metabolic dysfunction.

Because CD248 regulates many of these processes, including extracellular matrix remodeling, adipocyte function, and insulin signaling, we hypothesized that CD248 may represent a glucocorticoid-responsive component of adipose tissue remodeling in obesity. Therefore, we investigated the association between adipose tissue CD248 expression and obesity-related clinical features, characterized its expression across sexes and adipose depots, and examined whether CD248 expression is regulated by glucocorticoid stimulation.

## Materials and methods

### Study cohorts

#### Primary and T2D cohort

Abdominal subcutaneous adipose tissue (SAT) was obtained by needle aspiration after administration of local anesthetic lidocaine (Xylocain; AstraZeneca, Sweden) in the lower two quadrants of the abdomen of individuals without (75 male/114 female, age: 18–74 years; body mass index (BMI): 20.1–47.9 kg/m^2^) or with T2D according to World Health Organisation (WHO) criteria (21 male/33 female, age: 31–82 years; BMI: 22.4–42.4 kg/m^2^). Adipose tissue was snap frozen in liquid nitrogen. Due to limited sample availability, not all analyses were performed in all subjects. mRNA (*n* = 186) and protein (*n* = 40) analyses were performed in the primary cohort. Details on clinical parameters for the primary and T2D cohort are presented in Table 1, while sex-stratified data for the primary cohort can be found in Supplementary File S1.

#### Weight loss cohort

As previously described (Almby et al. 2021, Katsogiannos et al. 2019, Katsogiannos et al. 2020), individuals with T2D (2 male/9 female, age: 18–60 years, BMI: 30–45 kg/m²) underwent Roux-en-Y gastric bypass (RYGB) surgery at Uppsala University Hospital, Sweden. The participants were followed longitudinally, with samples collected at baseline prior to surgery and at 4, 24, and 104 weeks after surgery. A healthy non-diabetic control group was included for comparison, matched to the RYGB group at 104 weeks post-surgery (3 male/6 female, age: 49–68 years, BMI: 23.2–31.5 kg/m^2^). Adipose tissue was collected at each time point, followed by mRNA isolation and transcriptomic analyses with a focus on *CD248* expression.

#### Surgical cohort

40 healthy kidney donors (20 male/20 female, age: 30–71 years, BMI: 20.0–36.2 kg/m^2^) and 23 individuals without T2D undergoing bariatric surgery (4 male/19 female, age: 22–51 years, BMI: 33.0–54.4 kg/m^2^) from whom paired samples of SAT and omental adipose tissue (OAT) were obtained during surgery at Uppsala University Hospital or Sahlgrenska University Hospital. Samples were used for transcriptomics (*n* = 60) and protein analysis (*n* = 7). For details on clinical parameters, see Supplementary File S1.

**Table 1:** Clinical characteristics of study participants in the primary and type 2 diabetes cohorts

Clinical Parameters	No Diabetes			T2D
	Lean	Overweight	Obese	
Sex, females, n (%)	38 (63)	43 (57)	33 (61)	33 (61)
Age (years)	46 ± 18	56 ± 15^A^	50 ± 17	58 ± 10
BMI (kg/m^2^)	22.8 ± 1.26	27.3 ± 1.43^A^	34.0 ± 3.70^B, C^	32.5 ± 5.15
WHtR	0.49 ± 0.04	0.56 ± 0.04^A^	0.66 ± 0.06^B, C^	0.65 ± 0.07
WHR	0.86 ± 0.08	0.92 ± 0.08^A^	0.97 ± 0.10^B, c^	1.00 ± 0.07
Body fat %	25.0 ± 7.41	31.8 ± 7.36^A^	39.7 ± 8.30^B, C^	38.6 ± 8.48
Fasting glucose (mmol/L)	5.55 ± 0.48	5.81 ± 0.67^a^	5.87 ± 0.52^B^	8.28 ± 1.72
Fasting insulin (mU/L)	7.20 ± 4.62	8.71 ± 4.63	14.3 ± 6.91^B, C^	18.3 ± 9.53
HOMA-IR	1.80 ± 1.22	2.20 ± 1.19	4.20 ± 5.52^B, C^	6.40 ± 3.81
HbA1c (mmol/mol)	33.9 ± 3.08	35.0 ± 3.61	35.6 ± 3.61	52.0 ± 10.64
Adipocyte size SAT (µm)	94.0 ± 14.3	104.1 ± 11.6^A^	115.5 ± 8.4^B, C^	108.3 ± 13.0
Cholesterol (mmol/L)	4.92 ± 0.99	5.42 ± 1.06^A^	5.36 ± 0.98	4.69 ± 0.82
HDL-cholesterol (mmol/L)	1.51 ± 0.31	1.50 ± 0.36	1.32 ± 0.24^B^	1.13 ± 0.25
LDL-cholesterol (mmol/L)	3.00 ± 0.83	3.34 ± 0.95	3.46 ± 0.84^B^	2.92 ± 0.68
Triglycerides (mmol/L)	0.90 ± 0.40	1.20 ± 0.62^A^	1.50 ± 0.85^B^	1.71 ± 0.85

Note: Data are shown as mean ± standard deviation. Superscript letters indicate pairwise comparisons. a, A: lean vs. overweight; b, B: lean vs. obese; c, C: overweight vs. obese. Lower case: *p* < 0.05; Uppercase: *p* < 0.01

Abbreviations: T2D, Type 2 Diabetes; BMI, Body Mass Index; WHtR, Waist to Height ratio; WHR, Waist-Hip Ratio; HOMA-IR, Homeostatic Model Assessment of Insulin Resistance; HbA1c, Glycated Hemoglobin; HDL, High-Density Lipoprotein; LDL, Low-Density Lipoprotein

Anthropometric measurements were obtained from all individuals. BMI categories were defined as lean (< 25 kg/m^2^), overweight (25–29.9 kg/m^2^), and obese (≥ 30 kg/m^2^). Fasting blood samples were collected for analysis of plasma glucose, insulin and lipids at the Department of Clinical Chemistry, Uppsala or Sahlgrenska University Hospitals. Participants in the primary cohort were excluded if they had an ongoing or recent serious illness, such as endocrine disorders, cancer, cardiac events, or other major diseases, or any condition that could interfere with participation or safety. T2D was not considered an exclusion criterion in the T2D and weight loss cohort, and all individuals were treated with metformin only. Participants on medication that could influence the results, such as insulin, systemic glucocorticoids, anticoagulants, or immune-modulating therapies, were also excluded.

The study was approved by the Regional Ethics Review Boards in Uppsala (Dnr2013/330, Dnr2013/183/494, Dnr2018/385, Dnr2020/06350, and Dnr2024-07570-02) and Gothenburg (Dnr336-07, T508-9). All participants gave their written informed consent, and the reported investigations have been carried out in accordance with the principles of the Declaration of Helsinki as revised in 2008.

### Isolation of adipocytes and SVF

To obtain adipocytes and SVF, adipose tissue was digested with collagenase type II from *Clostridium histolyticum* (Sigma Chemical, St. Louis, MO) in Hank’s medium (Invitrogen Corporation, Paisley, UK) containing 6 mmol/L glucose, 4% bovine serum albumin (BSA, Sigma), 0.15 µmol/L adenosine (Sigma), pH 7.4, for 30–60 min at 37 °C in a shaking water bath. Following digestion, the cell suspension was filtered through a 250 μm nylon mesh. The floating adipocytes were collected and washed four times in Hank’s medium. The SVF was isolated from the medium by centrifugation for 3 min at 1200 rpm, followed by treatment with red blood cell lysis buffer (0.154 M NH₄Cl, 10 mM KHCO₃, and 0.1 mM EDTA (Sigma)) for 10 min. The cells were then washed with PBS, with centrifugation between steps.

Additionally, mature adipocytes were isolated for measurement of adipocyte size. Adipocyte size was determined by measuring the diameter of 100 individual cells per subject using brightfield microscopy of a monolayer of adipocytes.

### Adipose tissue and cell incubations

The clinical characteristics of participants included in the adipose tissue and cell incubation experiments with cortisol, dexamethasone, and estrogen are presented in Supplementary File S1. These experiments were performed to determine the response of CD248 expression to these treatments in adipose tissue and adipose tissue cell fractions.

#### Cortisol dose-response in adipose tissue

SAT (~ 100 mg) from 2 male and 3 female individuals (age: 23–71 years, BMI: 23.7–30.1 kg/m^2^) was incubated for 24 h at 37 °C and 5% CO₂ in DMEM containing 6 mmol/L glucose (Invitrogen Corporation, Paisley, UK), 10% fetal bovine serum (FBS, Invitrogen), and 1% penicillin–streptomycin (PEST, Invitrogen) with or without cortisol (10-4000 nM). To compare glucocorticoid responses, tissues were also incubated with dexamethasone, a potent and selective glucocorticoid receptor agonist commonly used as an alternative to cortisol in glucocorticoid stimulation experiments. Dexamethasone was used at 40 and 200 nM in the initial cortisol–dexamethasone comparison experiment and at 300 nM in subsequent depot-comparison and cell-fraction experiments. The 40 nM concentration was selected to approximate the potency of 1000 nM cortisol based on the relative glucocorticoid potency of dexamethasone (Liu et al. 2013), while 200 nM was included as a higher concentration approaching the 300 nM used in subsequent experiments. The 300 nM concentration was selected based on previous studies reporting robust glucocorticoid responses in human adipose tissue (Pereira et al. 2014, Lundgren et al. 2004). Following incubation, tissue samples were snap frozen and mRNA was isolated.

#### Dexamethasone response in adipose tissue depots

Depot-specific effects were assessed in SAT and OAT (~ 100 mg) from 4 male and 6 female individuals (age: 30–60 years, BMI: 22.6–29.2 kg/m^2^). Tissue samples were incubated for 24 h at 37 °C and 5% CO₂ in DMEM containing 6 mmol/L glucose, 10% FBS, and 1% PEST, with or without 300 nM dexamethasone, after which samples were snap frozen and mRNA was isolated.

#### Dexamethasone response in adipose tissue and cell fraction

Needle biopsy-derived SAT from 1 male and 7 female individuals (age: 30–82 years, BMI: 26.1–35.6 kg/m^2^) was incubated with or without dexamethasone (300 nM) using intact tissue, isolated adipocytes, and SVF cells to determine the cellular contribution to the glucocorticoid response. For these experiments, approximately 100 mg of intact adipose tissue, 200 µl of isolated adipocytes, or the isolated SVF cells were incubated for 24 h at 37 °C and 5% CO₂ in DMEM containing 6 mmol/L glucose, 10% FBS, and 1% PEST. After incubation, samples were snap frozen, and both mRNA and protein were isolated.

#### Estrogen response in adipose tissue

To assess the effect of estrogen on CD248 expression, SAT (*n* = 5 females, age: 21–79 years, BMI: 22.7–30.6 kg/m^2^) was incubated with 17β-estradiol (E2, Sigma) 1 nM for 24 h under the same culture conditions as previously reported (Kamble et al. 2019). Following incubation, tissue samples were snap-frozen and processed for mRNA analysis.

### Gene expression and transcriptomics

Total RNA was isolated from adipose tissue samples using the RNeasy Lipid Tissue Mini Kit (Qiagen, Hilden, Germany). RNA concentration and purity were assessed using a Nanodrop 2000 spectrophotometer (Thermo Fisher Scientific). Purified RNA (400 ng) was reverse transcribed into complementary DNA (cDNA) using the High-Capacity cDNA Reverse Transcription Kit (Applied Biosystems, Thermo Fisher Scientific), following the manufacturer’s instructions.

Gene expression was quantified by qPCR using TaqMan gene expression assays (Thermo Fisher) for *CD248* (Hs00535586). Amplification was performed on a QuantStudio 3 sequence detection system (Applied Biosystems). Relative gene expression levels were calculated using the 2∧-delta CT (ΔCt) method and normalized to beta-glucuronidase (GUSB, Hs00939627_m1). GUSB expression did not differ significantly between the groups, supporting its use as a reference gene. For stromal vascular fraction samples, glyceraldehyde-3-phosphate dehydrogenase (GAPDH, Hs02786624_g1) was used as the reference gene.

For transcriptomics analysis, samples of the weight loss cohort and surgical cohort were used for RNA sequencing at Novogene Co., Ltd. (Cambridge, UK). For the weight loss cohort, RNA-seq data were used to investigate changes in *CD248* gene expression after bariatric surgery. For the surgical cohort, expression data were normalized and corrected for technical batch effects, with BMI included as a covariate to preserve BMI-associated biological variation. To identify genes co-expressed with *CD248*, Pearson correlation coefficients were calculated within each depot. All genes were ranked by their Pearson correlation coefficient without applying a correlation cutoff and subjected to gene set enrichment analysis (GSEA) using MSigDB Hallmark gene sets (Subramanian et al. 2005). A normalized enrichment score (NES) was obtained for each pathway. Weighted gene co-expression network analysis (WGCNA) was performed separately for each depot to identify CD248-associated modules. Identified modules were subjected to Gene Ontology Biological Process enrichment analysis. Pathway and module enrichment significance was defined as Benjamini-Hochberg FDR < 0.05.

### Protein isolation and western blot

Adipose tissue or cells were homogenized in ice-cold lysis buffer (25 mmol/L Tris–HCl pH 7.4, 0.5 mmol/L EGTA, 25 mmol/L NaCl, 1% Nonidet P-40, 1 mmol/L Na_3_VO_4_, 10 mmol/L NaF, and 100 nmol/L okadaic acid), supplemented with 1x protease inhibitor cocktail (Roche), and lysed at 4 °C for 2 h. Lysates were centrifuged at 12,000 x g for 15 min at 4 °C, and the supernatant was collected and stored at − 80 °C. Protein concentration was measured using the BCA protein assay kit (Thermo Scientific, Rockford, IL).

Proteins (5–10 µg/lane) were separated by SDS-PAGE using Mini-PROTEAN TGX stain-free gels (Bio-Rad), transferred to low-fluorescence PVDF (Bio-Rad), and immunoblotted with antibodies against CD248 (1:1000, Proteintech, 60170-1-Ig). For CD248, the manufacturer reports a predicted molecular weight of ~ 80 kDa and an observed molecular weight of ~ 150 kDa. In line with its known glycosylation (Rettig et al. 1992), these bands were interpreted as the unglycosylated (~ 80 kDa) and glycosylated (~ 150 kDa) forms, respectively. Membranes were then incubated with a horseradish peroxidase-linked secondary antibody (1:2000, Cell Signaling, #7074S). Detection was performed using chemiluminescence reagent (ECL, Amersham Biosciences GE Healthcare) and a ChemiDoc XRS detection system (Bio-Rad). Bands and stain-free blots were quantified using Quantity One Imaging software (version 4.6.6 from Bio-Rad). Total protein expression data corresponding to these analyses are provided in Supplementary File S2.

### Circulating CD248 in UKBB

Proteomic profiling from the UK Biobank Pharma Proteomics Project from Olink proteomics data together with clinical and anthropometric measurements was used to extract circulating CD248 levels. Plasma proteins were quantified in UK Biobank participants using the Olink Explore 3072 platform, using proximity extension assay (PEA) technology coupled with next-generation sequencing for high-throughput measurement of approximately 3,000 proteins. Protein abundance was reported as normalized protein expression (NPX) values on a log2 scale following Olink quality control and normalization procedures. The analysis included 44,545 participants without diabetes. Individuals were stratified into BMI categories lean (< 25 kg/m^2^, *n* = 14,536, BMI: 22.8 ± 1.7 kg/m^2^, age: 56.0 ± 8.3 years), overweight (25–29.9 kg/m^2^, *n* = 19,112, BMI: 27.3 ± 1.4 kg/m^2^, age: 57.3 ± 8.2 years), and obese (≥ 30 kg/m^2^, *n* = 10,897, BMI: 34.0 ± 3.9 kg/m^2^, age: 57.2 ± 8.0 years). Circulating CD248 levels (NPX) were compared across BMI categories in the full cohort and separately in males and females.

To assess whether the association between HbA1c and circulating CD248 was independent of demographic and adiposity-related factors, multivariable linear regression was performed with circulating CD248 NPX as the dependent variable and HbA1c, age, sex, and BMI as independent variables. Analyses included participants with complete data for all variables (*n* = 42,163). Sex was coded as female versus male, with male as the reference category. Heteroscedasticity-consistent HC3 robust standard errors were used to calculate 95% confidence intervals and p-values. Analyses were performed in Python 3.11.15 using statsmodels v0.14.5.

UK Biobank data were accessed under application reference 217856. Data from UK Biobank participants were obtained with informed consent under the Human Tissue Act 2004, with ethical approval from the North West-Haydock Research Ethics Committee (REC reference 21/NW/0157; IRAS project ID 299116).

### Statistics

Data are presented as mean ± SEM unless otherwise stated. Normality was assessed using the Shapiro–Wilk test. Group comparisons were performed using Kruskal–Wallis tests or Mann–Whitney U tests for non-parametric data and one-way ANOVA or paired/unpaired t-tests for parametric data, as appropriate. Repeated measures within the same individuals were analysed using repeated measures two-way ANOVA or Friedman tests depending on data distribution. Associations between variables were assessed using Spearman correlation, and independent predictors were identified using multiple linear regression. The dose response curve was analysed using four-parameter logistic (4PL) non-linear regression model.

Multiple testing was controlled using the two-stage step-up method of Benjamini, Krieger, and Yekutieli to control the false discovery rate. For UK Biobank analyses, circulating CD248 levels were compared across BMI categories using one-way ANOVA and Kruskal–Wallis tests, with pairwise differences assessed by using Welch’s t-tests and effect sizes quantified by Cohen’s d. Associations between circulating CD248 levels and metabolic parameters were assessed using Pearson and Spearman correlation analyses in the full cohort and separately in males and females. Bonferroni correction was applied to control for multiple comparisons. Statistical analyses were performed using GraphPad Prism version 11.0.2 and SPSS version 30.0.0.0. Analyses of RNA sequencing data from the surgical cohort were performed in R version 4.5.0. A p-value < 0.05 was considered statistically significant, indicated as ∗ (*p* < 0.05), ∗∗ (*p* < 0.01), and ∗∗∗ (*p* < 0.001).

## Results

### CD248 expression in adipose tissue

**Fig. 1:**
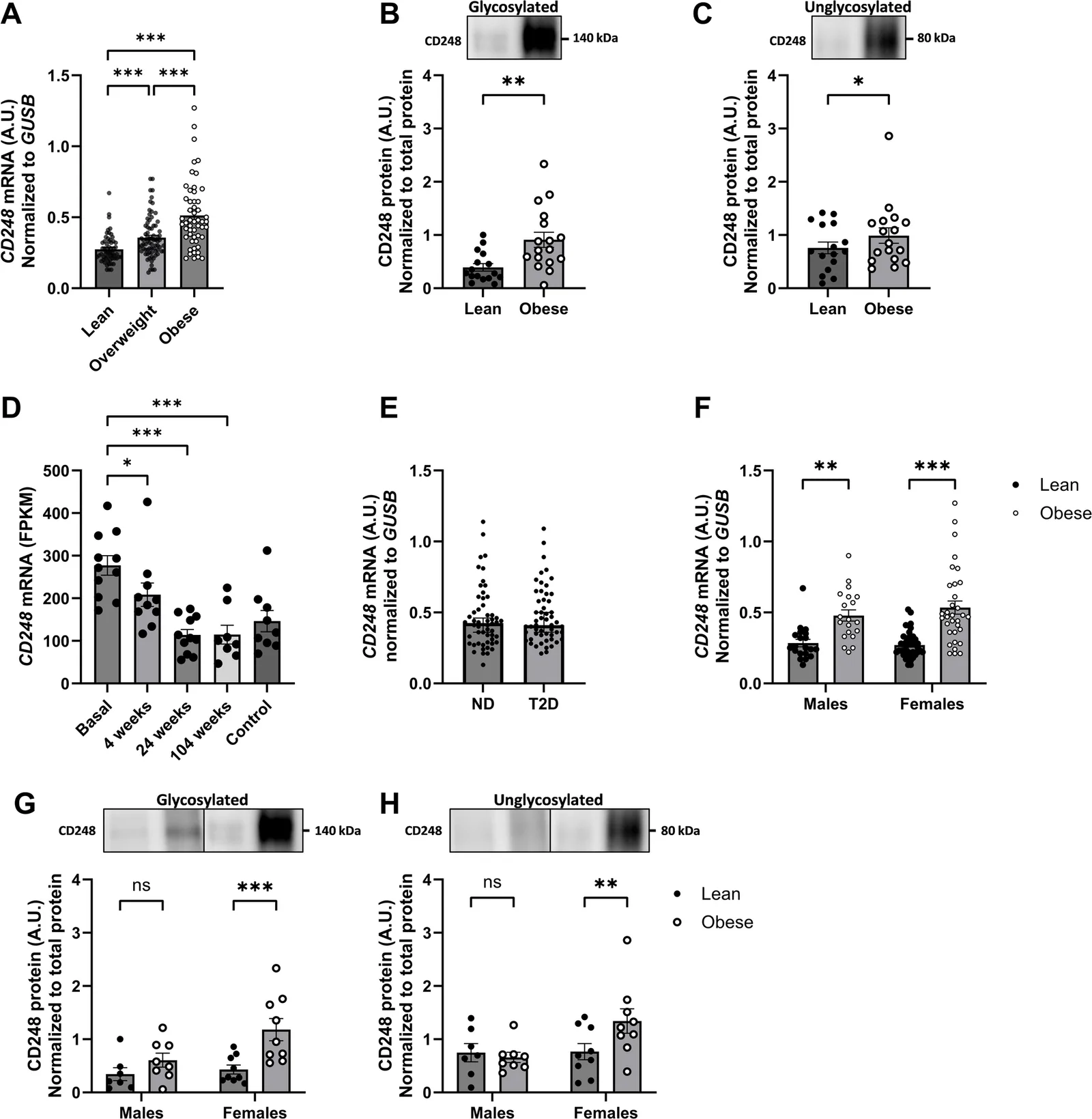
CD248 gene and protein expression from needle biopsies across different body mass index (BMI) groups in subcutaneous adipose tissue (SAT). (**A**) Gene expression levels of CD248 in lean (*n* = 58), overweight (*n* = 75), and obese (*n* = 53) individuals are shown in SAT. (**B**) Glycosylated CD248 protein levels in SAT are compared between lean (*n* = 16) and obese (*n* = 17) individuals. (**C**) Unglycosylated CD248 protein levels in SAT are shown for lean and obese individuals. Total protein expression data corresponding to these analyses are provided in Supplementary File S2. (**D**) CD248 mRNA expression in SAT, expressed as fragments per kilobase million reads (FPKM), was measured by transcriptomics in a weight loss cohort undergoing bariatric surgery (2 males, 9 females) at baseline and at 4, 24, and 104 weeks post-surgery, with a control group (3 males, 6 females). (**E**) CD248 gene expression in SAT comparing individuals with no diabetes (ND) and type 2 diabetes (T2D) (21 males and 33 females per group). Data are shown as median with 95% CI. (**F**) CD248 gene expression in SAT stratified by sex is presented for lean (20 males, 38 females) and obese individuals (20 males, 33 females). Data are shown as median with 95% CI. (**G**) Glycosylated CD248 protein levels in SAT stratified by sex are shown for lean (7 males, 9 females) and obese individuals (8 males, 9 females). (**H**) Unglycosylated CD248 protein levels in SAT stratified by sex are shown for lean and obese individuals

CD248 expression was measured in SAT from the primary cohort at both the gene and protein level to characterize its expression across BMI groups. *CD248* mRNA expression was significantly (*p* < 0.001) higher (~ 32%) in individuals with overweight compared with lean individuals and was further increased in individuals with obesity compared with both lean (~ 86%) and overweight groups (Fig. 1A). At the protein level, both glycosylated and unglycosylated CD248 were significantly (*p* < 0.05) increased in individuals with obesity compared with lean individuals (Fig. 1B, C). In the weight loss cohort, *CD248* mRNA expression decreased significantly (~ 25%) 4 weeks after bariatric surgery and reached levels comparable to controls by week 24 (Fig. 1D).

To assess whether *CD248* expression is associated with T2D status independent of adiposity, expression levels were compared between individuals with and without T2D, matched for BMI, age, and sex. Clinical characteristics of the matched groups are provided in Supplementary File S1. No significant difference was observed between the groups (Fig. 1E). In an additional transcriptomic analysis of 12 individuals with and 12 without T2D matched for BMI, age, and sex, no difference in CD248 expression was observed between the groups (Supplementary File S2).

Sex-stratified analysis revealed that, in both males and females, obese individuals had higher *CD248* expression compared with lean individuals (Fig. 1F). In a linear regression including sex, BMI, and their interaction, the sex x BMI interaction was not significant (F = 0.62, *p* = 0.431) and sex showed no independent association with *CD248*, confirming that the BMI-*CD248* association did not differ between males and females. In contrast, glycosylated and unglycosylated CD248 protein levels were significantly higher in females with obesity compared with lean females, whereas we did not find a difference between lean and obese males (Fig. 1G, H). To investigate whether sex hormones could contribute to these differences, adipose tissue was incubated with estrogen for 24 h, which increased *CD248* mRNA expression by 36% (*p* < 0.01) (Supplementary File S2).

### CD248 expression and clinical parameters

We further explored the association of CD248 with clinical parameters (Table 2). *CD248* gene expression in adipose tissue from the primary cohort was positively associated with markers of adiposity, including BMI (rho = 0.503, *p* < 0.001) and waist to height ratio (WHtR, rho = 0.514, *p* < 0.001). In addition, *CD248* gene expression correlated with markers of metabolic dysfunction, including fasting insulin (rho = 0.417, *p* < 0.001), HOMA-IR (rho = 0.386, *p* < 0.001), triglycerides (rho = 0.287, *p* < 0.001) and the LDL/HDL ratio (rho = 0.388, *p* < 0.001). CD248 protein followed the same pattern, correlating with CD248 mRNA (rho = 0.605, *p* < 0.001) and showing positive associations with BMI (rho = 0.520, *p* < 0.001), WHtR (rho = 0.454, *p* = 0.003), fasting insulin (rho = 0.3121, *p* = 0.043), and triglycerides (rho = 0.386, *p* = 0.014) (Supplementary File S1).

**Table 2:** Correlations between CD248 gene expression in subcutaneous adipose tissue (SAT) and clinical parameters

Clinical parameter	No Diabetes			T2D		
	rho	*p*	*n*	rho	*p*	*n*
Age	0.090	0.221	186	-0.213	0.122	54
Sex (F vs. M)	-0.007	0.929	186	**0.398**	**0.003**	54
BMI	**0.503**	**< 0.001**	186	0.157	0.258	54
Waist	**0.478**	**< 0.001**	185	-0.123	0.376	54
WHR	**0.294**	**< 0.001**	184	-0.258	0.059	54
WHtR	**0.514**	**< 0.001**	185	0.201	0.146	54
Body fat %	**0.370**	**< 0.001**	165	**0.486**	**0.002**	37
Fasting glucose	**0.150**	**0.042**	184	0.069	0.620	54
Fasting insulin	**0.417**	**< 0.001**	186	**0.315**	**0.023**	52
C-Peptide	**0.472**	**< 0.001**	184	0.186	0.191	51
HOMA-IR	**0.386**	**< 0.001**	184	0.256	0.067	52
HbA1c	**0.176**	**0.017**	184	-0.086	0.538	54
Total cholesterol	**0.216**	**0.003**	185	-0.158	0.252	54
Triglycerides	**0.287**	**< 0.001**	186	0.063	0.650	54
HDL - cholesterol	**-0.240**	**< 0.001**	186	-0.172	0.213	54
LDL - cholesterol	**0.310**	**< 0.001**	186	-0.138	0.321	54
LDL/HDL	**0.388**	**< 0.001**	186	-0.007	0.959	54
Adipocyte size	**0.399**	**< 0.001**	127	0.209	0.167	45

Note: Correlations are Spearman. Bolded values indicate correlations *p*<0.05

Abbreviations: T2D, Type 2 Diabetes; F, Female; M, Male; BMI, Body Mass Index; WHR, Waist-Hip Ratio; WHtR, Waist-to-Height Ratio, HOMA-IR, Homeostatic Model Assessment of Insulin Resistance; HbA1c, Glycated Hemoglobin; HDL, High-Density Lipoprotein; LDL, Low-Density Lipoprotein.

Within the T2D cohort, *CD248* gene expression was positively correlated with sex (rho = 0.398, *p* = 0.003), body fat % (rho = 0.486, *p* = 0.002), and insulin (rho = 0.315, *p* = 0.023), while HOMA-IR showed a trend to significance (rho = 0.256, *p* = 0.067) (Table 2).

Multiple regression analysis in the primary cohort was performed to identify independent predictors of *CD248* mRNA expression (Table 3). After adjustment for sex, age, and WHR, both BMI (std β = 0.345, *p* < 0.001) and fasting insulin (std β = 0.206, *p* = 0.022) were significant predictors (R²= 0.227, *p* < 0.001) of *CD248* mRNA expression. When fasting insulin and glucose were replaced with HOMA-IR, only BMI remained significant (std β = 0.444, *p* < 0.001, R²=0.199, *p* < 0.001). A parallel regression analysis for CD248 protein, including sex, age, BMI, and HOMA-IR, confirmed BMI as an independent predictor, similar to the mRNA model (Supplementary File S1). In contrast to mRNA, sex was also an independent predictor, with higher protein expression in females.

**Table 3:** Linear regression models between CD248 gene expression in subcutaneous adipose tissue and clinical parameters in individuals with or without type 2 diabetes

Model	Clinical parameters	No Diabetes		T2D	
		Std β	*p*	Std β	*p*
1	Sex (F vs. M)	0.052	0.535	**0.383**	**0.019**
	Age	0.067	0.431	-0.271	0.070
	BMI	**0.345**	**< 0.001**	-0.249	0.156
	WHR	-0.005	0.962	0.002	0.988
	Fasting insulin	**0.206**	**0.022**	0.260	0.120
	Fasting glucose	-0.126	0.107	0.092	0.489
R^2^		0.227	< 0.001	0.259	0.029
2	Sex (F vs. M)	0.083	0.328	**0.391**	**0.014**
	Age	-0.021	0.795	-0.278	0.062
	BMI	**0.444**	**< 0.001**	-0.219	0.196
	WHR	0.036	0.725	0.015	0.923
	HOMA-IR	-0.039	0.611	0.223	0.147
R^2^		0.199	< 0.001	0.240	0.023

Note: Linear regression model. Bolded values indicate significance *p* < 0.05. ND: 72 males/112 females. T2D: 19 males/33 females

Abbreviations: T2D, Type 2 Diabetes; F, Females; M, Males; Std β, Standardized Regression Coefficients; BMI, Body Mass Index; WHR, Waist-Hip Ratio; HOMA-IR, Homeostatic Model Assessment of Insulin Resistance

In a regression analysis restricted to individuals in the T2D cohort (Table 3), sex was the only significant predictor of *CD248* mRNA expression, with higher expression observed in females (std β = 0.383, *p* = 0.019; model R²=0.259, *p* = 0.029).

Potential sex differences in the primary cohort were evaluated by stratifying associations between *CD248* expression and clinical parameters (Supplementary File S1). In males, *CD248* expression was positively correlated with BMI (rho = 0.470, *p* < 0.001) and HOMA-IR (rho = 0.396, *p* < 0.001), whereas in females it correlated with HbA1c (rho = 0.212, *p* = 0.024), and menopausal status, with higher expression in postmenopausal compared with premenopausal females (rho = 0.229, *p* = 0.014).

In sex-stratified multiple regression analysis (Supplementary File S1), BMI was an independent predictor of *CD248* expression in both males (std β = 0.385, *p* = 0.018) and females (std β = 0.295, *p* = 0.015). In females, fasting insulin was an additional predictor (std β = 0.250, p = 0.049). Replacing insulin and glucose with HOMA-IR did not change the BMI association, and HOMA-IR itself was not significant (females: std β = 0.202, p = 0.072). Replacing menopausal status with age did not change these results.

### Comparison of depots

**Fig. 2:**
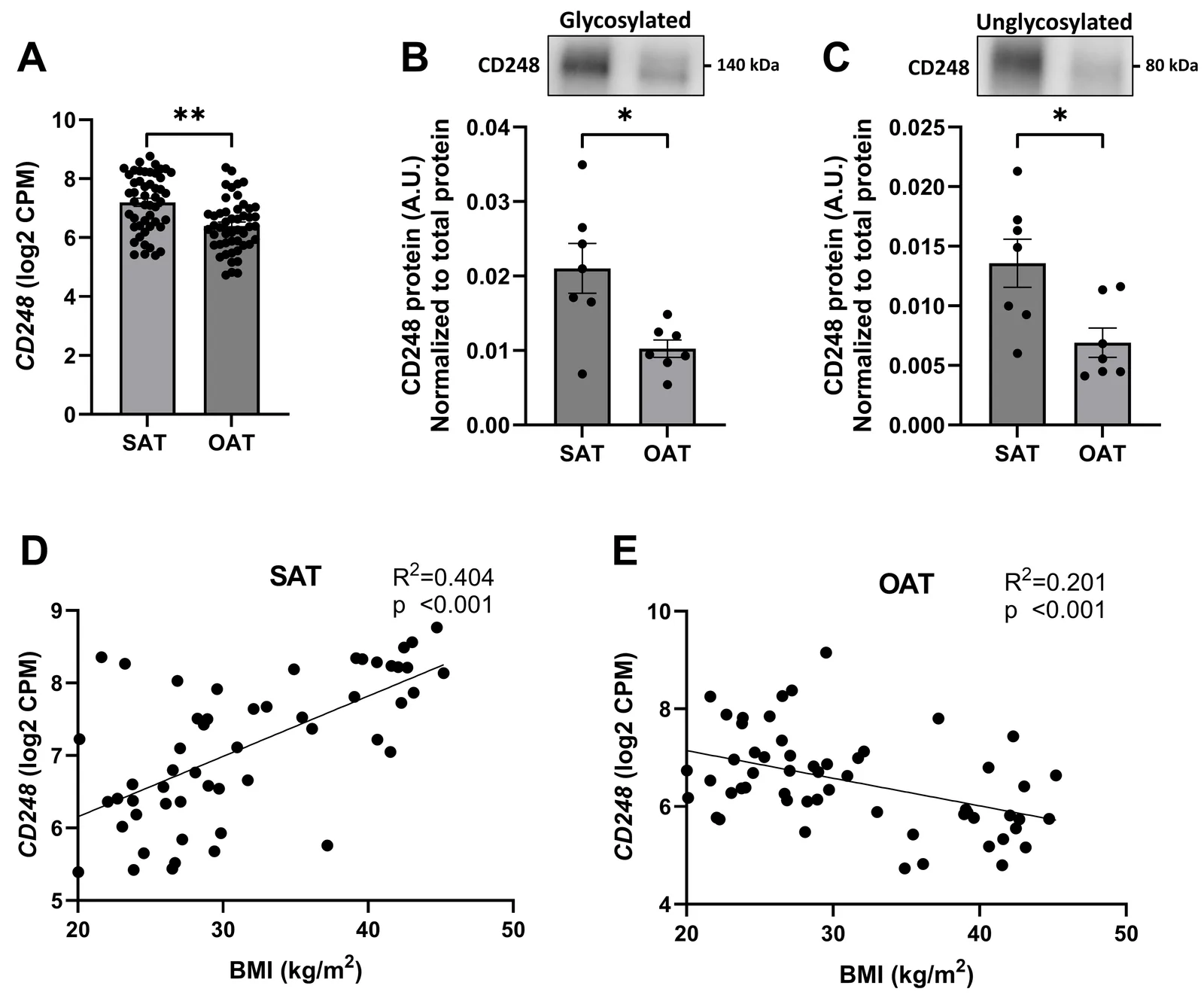
CD248 expression in subcutaneous and omental adipose tissue. Direct *CD248* mRNA comparison (*n* = 49) between the two tissues (**A**), followed by glycosylated (**B**) and unglycosylated (**C**) CD248 protein levels (*n* = 7) are shown for both SAT and OAT. Transcriptomic correlations between CD248 and BMI are shown for SAT (**D**) and for OAT (**E**) (*n* = 53 for SAT, *n* = 56 for OAT). Total protein expression data corresponding to these analyses are provided in Supplementary File S2

Samples from the surgical cohort were used to measure CD248 in both SAT and OAT, allowing direct comparison between the two adipose tissue depots. OAT exhibited significantly lower CD248 mRNA (*p* < 0.01) and protein levels (*p* < 0.05) compared with SAT (Fig. 2A-C). Furthermore, *CD248* expression in SAT was positively correlated with BMI (Fig. 2D), whereas in OAT it was negatively correlated with BMI (Fig. 2E).

To further characterize depot-specific patterns, we investigated associations between *CD248* expression and clinical parameters in both SAT and OAT (Supplementary File S1). In SAT, *CD248* gene expression was negatively associated with age (rho=-0.302, *p* = 0.03) and HDL cholesterol (rho=-0.504, *p* < 0.001), and positively associated with markers of adiposity including BMI (rho = 0.599, *p* < 0.001), and WHtR (rho = 0.598, *p* < 0.001), and markers of metabolic dysfunction including C-Peptide (rho = 0.564, *p* < 0.001), HOMA-IR (rho = 0.312, *p* = 0.026), and HbA1c (rho = 0.344, *p* = 0.013), and adipocyte size in both SAT (rho = 0.490, *p* < 0.001) and OAT (rho = 0.381, *p* = 0.017). In OAT, *CD248* gene expression was negatively associated with BMI (rho=-0.432, *p* < 0.001), WHtR (rho=-0.388, *p* = 0.004), and adipocyte size in SAT (rho=-0.350, *p* = 0.017), while a positive association was observed with HDL cholesterol (rho = 0.364, *p* = 0.007).

When age, sex, BMI, fasting insulin, and fasting glucose were entered into a multivariable regression model, BMI (std β = 0.424, *p* = 0.009) emerged as the only independent predictor of *CD248* expression in SAT (R²=0.320, *p* = 0.021), whereas no significant associations were observed in OAT (Supplementary File S1). When fasting insulin and fasting glucose were replaced with HOMA-IR, BMI (SAT: std β = 0.581, *p* < 0.001, OAT: std β=-0.406, *p* = 0.006) remained a significant independent predictor of *CD248* expression in both depots, although with opposite directions of association (SAT: R²=0.426, *p* < 0.001; OAT: R²=0.224, *p* = 0.013).

### Pathway analysis

**Fig. 3:**
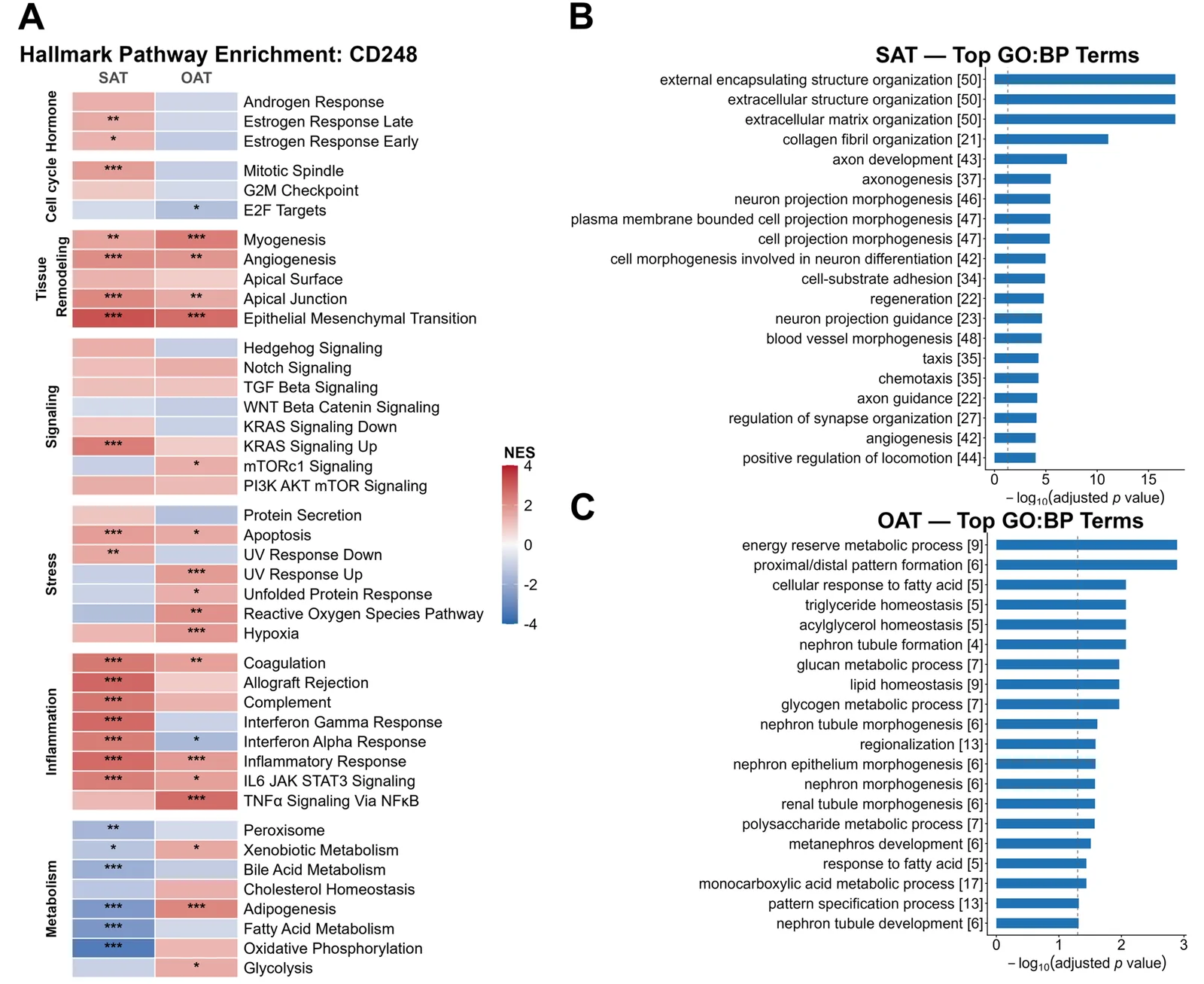
*CD248*-associated pathway analysis in subcutaneous and omental adipose tissue. Hallmark pathway enrichment of genes correlated with *CD248* based on normalized enrichment scores (NES) (**A**). Gene Ontology: Biological Process (GO: BP) enrichment of genes in the *CD248* associated Weighted Gene Co-expression Network Analysis (WGCNA) module in subcutaneous adipose tissue (SAT) (**B**) and in omental adipose tissue (OAT) (**C**). (*n* = 53 for SAT, *n* = 56 for OAT)

Gene set enrichment analysis of *CD248*-correlated genes revealed enrichment across multiple Hallmark pathways, including metabolic, inflammatory, stress-related, signalling, and cellular organization processes (Fig. 3A, Supplementary File S3).

In SAT, *CD248* expression was positively associated with inflammatory and remodelling-related pathways, including IL6-JAK-STAT3 signalling, complement, coagulation, and epithelial-mesenchymal transition. Additional enrichment was observed for pathways related to cellular stress and proliferation. In contrast, metabolic pathways such as oxidative phosphorylation, fatty acid metabolism, and adipogenesis were predominantly negatively associated with *CD248* expression.

In OAT, *CD248*-associated genes showed enrichment of inflammatory, stress-related, and remodelling pathways, including TNFA and IL6 signalling, hypoxia, reactive oxygen species, unfolded protein response, epithelial-mesenchymal transition, and angiogenesis. Metabolic pathways, including glycolysis and adipogenesis, were positively associated with *CD248* expression. Cell cycle related pathways (E2F targets) and interferon signalling showed negative associations, while mTORC1 signalling was specifically enriched in OAT.

To further investigate *CD248*-associated transcriptional networks, WGCNA was performed separately in SAT and OAT (Supplementary File S4). The top pathways identified in the *CD248* associated SAT module included extracellular matrix organization, collagen fibril organization, angiogenesis, cell adhesion, chemotaxis, and other tissue remodelling-related processes (Fig. 3B). In contrast, the top pathways identified in the *CD248*-associated OAT module included lipid homeostasis, fatty acid responses, glycogen metabolism, and developmental processes (Fig. 3C).

### Glucocorticoid regulation of CD248 expression

**Fig. 4:**
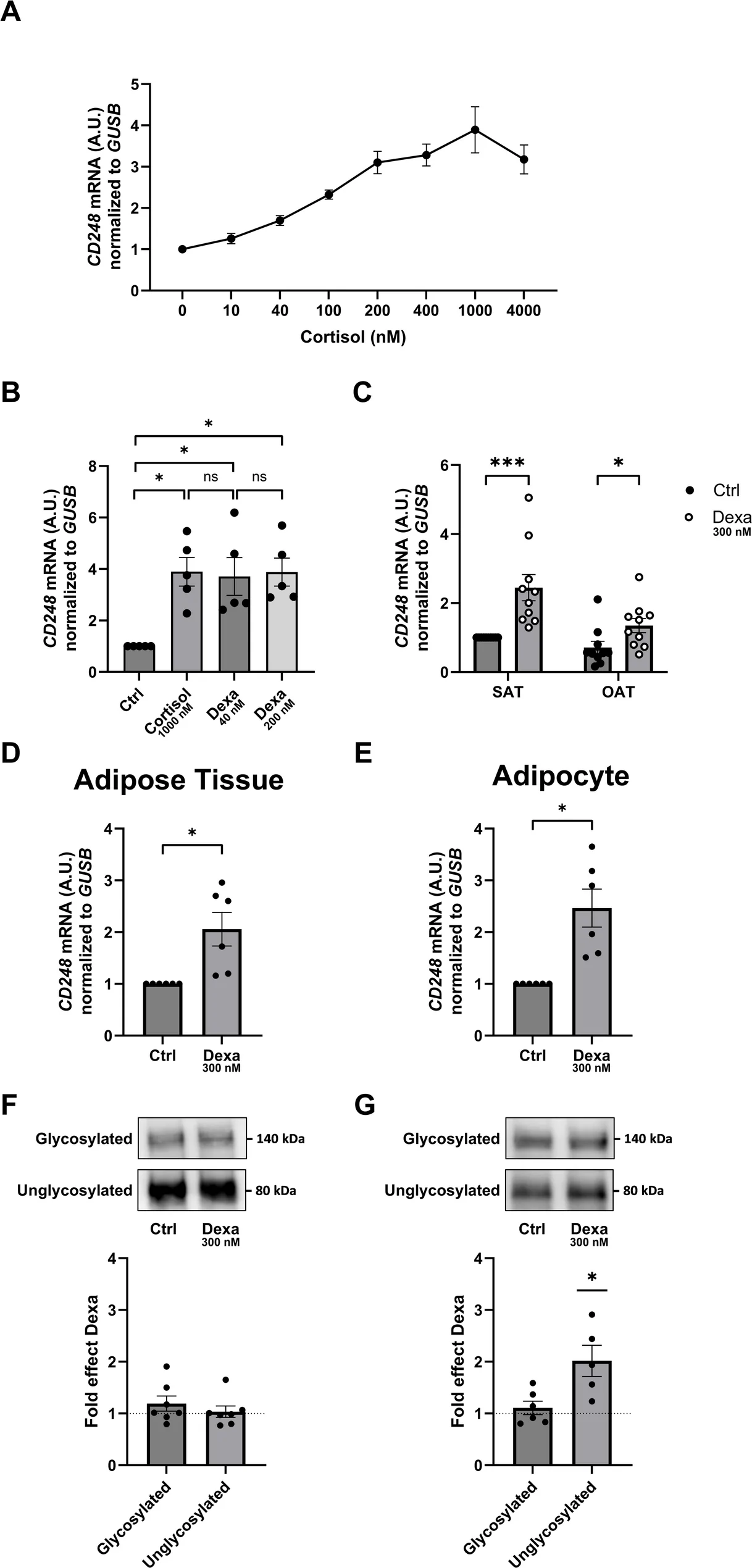
CD248 expression in adipose tissue and isolated adipocytes following 24-hour glucocorticoid stimulation. (**A**) *CD248* gene expression in subcutaneous adipose tissue (SAT) (2 males, 3 females) in response to increasing concentrations of cortisol (10, 40, 100, 200, 400, 1000, and 4000 nM). (**B**) *CD248* gene expression in SAT under control conditions and following cortisol (1000 nM) or dexamethasone (40 and 200 nM) treatment. (**C**) *CD248* gene expression after dexamethasone treatment in SAT and omental adipose tissue (OAT) (4 males/6 females). (**D**) *CD248* gene expression in adipose tissue and (**E**) isolated adipocytes following dexamethasone stimulation relative to control (1 male/7 females). Glycosylated and unglycosylated CD248 protein levels in (**F**) adipose tissue and (**G**) isolated adipocytes following dexamethasone stimulation. Total protein expression data corresponding to these analyses are provided in Supplementary File S2

To investigate whether CD248 is regulated by glucocorticoids, adipose tissue was examined following 24-hour dexamethasone or cortisol treatment. In a cortisol dose response assay, *CD248* expression showed a sigmoidal response with an EC50 of approximately 100 nM and maximal induction around 1000 nM (Fig. 4A). Dexamethasone at both 40 and 200 nM induced *CD248* expression to a similar magnitude as the maximal response observed with 1000 nM cortisol (Fig. 4B). *CD248* expression increased significantly in both SAT and OAT in response to dexamethasone (Fig. 4C), although OAT displayed lower basal expression, consistent with previous observations. The dexamethasone-induced fold-change in *CD248* expression was inversely correlated with BMI in SAT (rho=-0.64, *p* = 0.05), whereas no correlation was observed in OAT (rho=-0.0424, *p* = 0.92). To further assess the possibility of an early response, we examined publicly available transcriptomic data from SGBS adipocytes exposed to dexamethasone for 2 h (Balliu et al. 2021). No change in *CD248* expression was observed at this time point (Supplementary File S2).

Whole adipose tissue, isolated adipocytes, and SVF were incubated with dexamethasone to determine which cells contribute to CD248 expression. Dexamethasone significantly increased *CD248* mRNA expression in whole adipose tissue and isolated adipocytes (both *p* < 0.05) (Fig. 4D, E), whereas no response was observed in SVF (data not shown). At the protein level, CD248 expression in whole adipose tissue did not change following dexamethasone stimulation (Fig. 4F). In isolated adipocytes, glycosylated CD248 was also unchanged, whereas unglycosylated CD248 was significantly increased (*p* < 0.05, Fig. 4G).

### Circulating CD248 levels

**Fig. 5:**
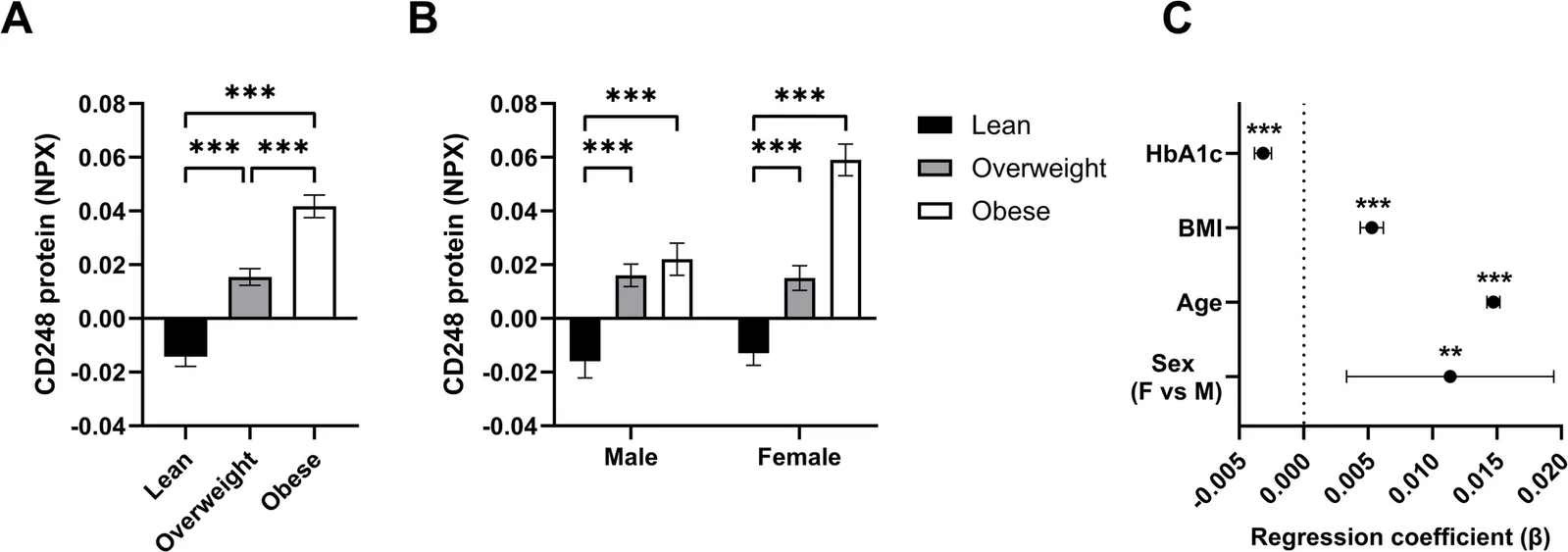
Circulating CD248 levels in the UKBB according to BMI category. (**A**) Circulating CD248 levels in lean (*n* = 14,536), overweight (*n* = 19,112) and obese (*n* = 10,897) participants are shown. (**B**) Circulating CD248 levels stratified by BMI and sex for lean (5,107 males/9,429 females), overweight (10,188 males/8,924 females), and obese (5,134 males/5,763 females) individuals. (**C**) Regression coefficients (β) and 95% confidence intervals from a multivariable linear regression model for the association of HbA1c, Body Mass Index (BMI), age and sex with circulating CD248 levels, adjusted for all covariates shown (*n* = 42,163)

Circulating CD248 protein levels were assessed in UK Biobank participants with both CD248 and BMI measurements (*n* = 44,545) to determine whether the BMI-associated increase observed in adipose tissue was also present systemically. CD248 levels were higher in overweight individuals than lean individuals and were further increased in participants with obesity compared with both lean and overweight groups; all pairwise Welch’s t-tests were significant at *p* < 0.001 (Fig. 5A).

When stratified by sex, the BMI-associated elevation in circulating CD248 protein was present in both males and females (one-way ANOVA, *p* < 0.001 for each sex (Fig. 5B)). The effect was more pronounced in females, with females with obesity showing approximately 2-fold greater effect size than males with obesity (Cohen’s d = 0.164 vs. 0.087) relative to their respective lean groups.

Unadjusted Spearman correlation analysis showed weak overall associations between circulating CD248 and BMI, glucose, HbA1c, WHR, and WHtR. The strongest correlation was observed with HbA1c (rho = 0.080, *p* < 0.001). The HbA1c correlation was stronger in females (rho = 0.130, *p* < 0.001) than in males (rho = 0.022, *p* = 0.003), although the male association was very small in magnitude. To determine whether the relationship between circulating CD248 and HbA1c was independent of demographic and adiposity-related factors, a multivariable linear regression analysis was performed adjusting for age, sex and BMI (Fig. 5C). HbA1c remained significantly associated with circulating CD248 (β=-0.0032 NPX per mmol/mol HbA1c; 95% CI -0.0038 to -0.0025; HC3-robust *p* < 0.001; *n* = 42,163). Notably, the direction of association was reversed after adjustment, indicating that the positive unadjusted correlation was substantially attenuated by age, sex, and particularly adiposity. These findings suggest that circulating CD248 is more strongly associated with obesity-related characteristics than with glycaemic status itself.

## Discussion

CD248 expression in adipose tissue is dynamically regulated in obesity, with differences between adipose depots, a sex effect at the protein level, and responsiveness to glucocorticoids. In the present study, we show that CD248 is altered in subcutaneous adipose tissue at both the mRNA and protein level. We further identify lower CD248 expression in omental compared with subcutaneous adipose tissue across both gene and protein levels, highlighting clear depot-specific variation. In addition, obesity was associated with markedly higher CD248 protein abundance in females, while no significant difference was observed in males. Finally, we demonstrate that CD248 responds to dexamethasone treatment, suggesting glucocorticoid mediated regulation of CD248 in adipose tissue.

CD248 increased with BMI in SAT at both the mRNA and protein level. While the increase in mRNA expression has been reported previously (Petrus et al. 2019), we additionally show that CD248 protein levels follow the same pattern. This is consistent with the known association of CD248 with fibrosis and hypoxia (Li et al. 2025, Ohradanova et al. 2008, Petrus et al. 2019), processes characteristic of expanding subcutaneous adipose tissue in obesity (Datta et al. 2018). In line with its association with adiposity, *CD248* levels decreased following bariatric surgery, returning to levels comparable to control at later time points. Previous studies have similarly reported reduced *CD248* levels in subcutaneous adipose tissue after bariatric surgery (Petrus et al. 2019), in some cases correlating with the extent of weight loss (Bouchard-Mercier et al. 2022), although this association was not observed in the present study, potentially due to limited sample size.

In multivariable regression models including measures of adiposity and insulin-related traits, BMI emerged as the strongest and mostly consistent predictor of *CD248* gene expression in subcutaneous adipose tissue. The lack of a similar association with measures of fat distribution suggests that *CD248* is more closely related to overall adiposity than regional fat distribution, with hyperinsulinemia contributing to a lesser extent. However, this does not exclude direct regulation by metabolic stimuli. Previous work has shown that glucose, insulin, and IGF-1 can alter *CD248* expression in SGBS adipocytes (Balliu et al. 2021), and similar metabolic regulation has been observed in other cellular models (Ku et al. 2025, Krishnan et al. 2023). In contrast, no significant difference in *CD248* mRNA levels was observed between individuals with or without T2D. In our primary cohort, BMI was the strongest and most consistent independent predictor of CD248 expression, whereas associations with insulin-resistance measures were attenuated after adjustment for BMI. These findings suggest that CD248 expression is more strongly related to adiposity than to diabetes status itself. This differs from work by (Petrus et al. 2019), who reported an association between *CD248* expression and diabetes in non-obese men with T2D. Differences in cohort characteristics, including sex, adiposity, diabetes duration, glycaemic control, and treatment, may contribute to the discrepant findings.

To account for potential sex-specific effects, the cohort was stratified by sex. Both males and females showed higher *CD248* gene expression in obesity compared with lean individuals, consistent with preclinical findings reporting no clear sex differences across adipose depots, with the exception of gonadal fat (Patrick et al. 2023). However, CD248 protein abundance was higher in females with obesity compared with lean females, a pattern not observed in males. Sex also remained an independent predictor of CD248 protein abundance after adjustment for BMI and HOMA-IR. In mice, sex-dependent effects of CD248 deficiency have been reported, although findings have not been consistent between studies (Patrick et al. 2023, Armitage et al. 2021). BMI remained the main predictor of *CD248* gene expression in both sexes, while in females insulin also emerged as an additional predictor. This may suggest a sex-specific association between CD248 and metabolic dysregulation in females. In line with a potential role of sex hormones in regulating CD248, our ex vivo experiments showed that estrogen increased *CD248* mRNA expression in adipose tissue. Further studies are needed to determine whether estrogen contributes to the sex-specific differences in CD248 protein levels and whether post-transcriptional or post-translational mechanisms are involved.

To assess whether the adipose tissue signal was reflected systemically, circulating CD248 levels were measured. Circulating CD248 increased across BMI categories, mirroring the pattern observed in SAT and with previous reports linking circulating CD248 to BMI (Anwar et al. 2026). The circulating form represents the soluble ectodomain of CD248, mostly generated by MMP-dependent cleavage of the membrane-bound protein (Payet et al. 2023) and previous work suggested that CD248 may be derived from SAT (Hruska et al. 2022). The parallel increase in SAT and circulating CD248 raises the possibility that adipose tissue contributes to circulating levels, although this requires further investigation. Notably, the positive association between HbA1c and circulating CD248 was attenuated and became inverse after adjustment for age, sex and BMI. Thus, the unadjusted association should not be interpreted as a direct relationship with glycaemic dysregulation. Although soluble CD248 has previously been shown to bind the insulin receptor and attenuate insulin-induced autophosphorylation (Benedet et al. 2023), the small, adjusted association observed here does not establish a functional or causal relationship with insulin signalling.

CD248 expression was higher in SAT compared with OAT, at both the transcript and protein level, consistent with previous large proteomic studies showing substantially higher protein levels in SAT than OAT, although these studies were performed in cohorts with severe obesity (Hruska et al. 2022, Hruska et al. 2023). Another proteomic study of visceral adipose tissue did not identify clear depot differences in CD248 (Dadson et al. 2026). Interestingly, the association between *CD248* gene expression and BMI differed between depots, with a positive association in SAT and a negative association in OAT. In OAT, this inverse association remained significant after adjustment for age, sex, and HOMA-IR. These opposing associations may reflect differences in hypoxia, extracellular matrix remodelling, or other regulatory processes between depots. In mice, adipocyte-specific loss of CD248 reduces hypoxia and fibrosis in visceral adipose tissue, linking CD248 to processes relevant to adipose tissue remodelling during obesity (Petrus et al. 2019). However, these processes do not appear to develop uniformly across adipose depots. Human studies indicate that the relationship between adipose tissue fibrosis and metabolic dysfunction differs between SAT and OAT (Datta et al. 2018, Seo et al. 2026), while transcriptomic studies have also demonstrated substantial depot-specific responses to obesity (Haffa et al. 2019, Ronquillo et al. 2019).

The pathway and network analyses further supported depot-specific associations of *CD248* mRNA. In SAT, genes co-expressed with *CD248* mRNA were enriched in extracellular matrix organization, angiogenesis, and cell adhesion, consistent with extracellular matrix remodelling that occurs in SAT expansion in obesity (Reyes-Farias et al. 2021) and with previous reports implicating CD248 in structural remodelling (Petrus et al. 2019). In OAT, *CD248*-associated genes were enriched in lipid metabolism, energy handling, and cellular stress responses, reflecting the metabolic characteristics of this depot (Reyes-Farias et al. 2021). Together, these findings suggest that *CD248* mRNA is associated with distinct transcriptional programs in SAT and OAT.

The enrichment of stress-responsive, inflammatory, and extracellular matrix remodelling pathways prompted us to investigate the glucocorticoid regulation of CD248. Dexamethasone increased *CD248* gene expression in adipose tissue, with an inverse association between BMI and the glucocorticoid response in SAT. This may reflect obesity-related changes in glucocorticoid receptor signaling, local cortisol metabolism (e.g., 11β-HSD1 activity), chromatin accessibility, inflammatory tone, or cell-type composition, all of which can influence glucocorticoid responses in human adipose tissue (Lee et al. 2014, Yu et al. 2010, Sandeep et al. 2005, Laterveer et al. 2026). The cortisol dose-response further supported glucocorticoid sensitivity, while the absence of an early response in human SGBS adipocytes suggests that *CD248* induction may not occur in the early phase of glucocorticoid exposure (Balliu et al. 2021). This is consistent with an independent human dexamethasone intervention study reporting a comparable approximately 1.6-fold increase in adipose tissue, with no corresponding regulation in skeletal muscle, suggesting a tissue-specific effect (Viguerie et al. 2012). Furthermore, a separate microarray study showed an increase in *CD248* mRNA expression following dexamethasone exposure, supporting the direction of change observed in our data (Pereira et al. 2014). The response in isolated adipocytes suggests that adipocytes are an important contributor to the regulation observed in whole adipose tissue.

The mechanisms underlying glucocorticoid-induced CD248 upregulation remain unclear. Glucocorticoid receptor signalling may interact with transcriptional cofactors such as SP1, while hypoxia-related pathways such as HIF-2α or TGFβ signalling represent additional potential mechanisms (Lu et al. 2024, Ohradanova et al. 2008, Suehiro et al. 2004, Ijichi et al. 2009, Suresh Babu et al. 2014, Shin et al. 2003). Interestingly, total CD248 protein levels did not increase, although the unglycosylated form of CD248 protein increased in adipocytes, suggesting possible post-translational regulation. The absence of detectable changes in total protein levels may relate to potential shedding of CD248 from the cell surface, as described previously. The response of *CD248* gene expression to both glucocorticoids and estrogen raises the possibility of an interaction between these signalling pathways, as previously described for other genes in human adipose tissue (Kamble et al. 2019).

This study has several limitations. In female participants, the lack of information on menstrual cycle phase may affect the generalizability of the findings, as hormonal fluctuations can influence adipose tissue function. In addition, although all samples were collected in the morning to reduce diurnal variation, residual differences in sampling time may still have influenced gene expression and metabolic measures, given the known time-dependent variation in adipose tissue biology. Within the surgical cohort, only the bariatric participants had followed a preoperative dietary intervention, which may have influenced adipose tissue characteristics and gene expression independently of adiposity. The dexamethasone experiments were primarily conducted in female-derived cells, which may influence the interpretation of glucocorticoid responsiveness across sexes. Finally, limited sample availability meant that not all analyses could be performed in every subject.

In summary, CD248 is upregulated in obesity and associated with adiposity-related metabolic disturbances, particularly in subcutaneous adipose tissue, and its expression is reversible following bariatric surgery. In adipose tissue, CD248 is induced by glucocorticoids, particularly in adipocytes, and its expression is associated with markers of insulin resistance, adiposity, and inflammatory and extracellular matrix remodelling pathways. At the systemic level, circulating CD248 was likewise elevated in obesity and correlated with HbA1c, an association that became inverse after adjustment for age, sex, and BMI. Collectively, these findings identify CD248 as an obesity associated factor at both the adipose tissue and systemic levels that is upregulated by glucocorticoids, and, given its previously described inhibitory coupling to the insulin receptor, support a possible role for CD248 in glucocorticoid- and obesity-related insulin resistance, warranting further mechanistic investigation.

## Supplementary Information

Below is the link to the electronic supplementary material.

Supplementary Material 1

Supplementary Material 2

Supplementary Material 3

Supplementary Material 4

## Data Availability

Data are not publicly available but can be provided from the corresponding author upon reasonable request.

## Abbreviations

BMI: Body Mass Index
cDNA: complementary DNA
Ctrl: Control
Dexa: Dexamethasone
F: Female
GAPDH: Glyceraldehyde-3-phosphate dehydrogenase
GUSB: Glucuronidase beta
HbA1c: Glycated hemoglobin
HDL: High-Density Lipoprotein
HOMA-IR: Homeostatic Model Assessment of Insulin Resistance
LDL: Low-Density Lipoprotein
M: Male
ND: No Diabetes
NPX: Normalized Protein Expression
OAT: Omental Adipose Tissue
PEA: Proximity Extension Assay
RYGB: Roux-en-Y Gastric Bypass
SAT: Subcutaneous Adipose Tissue
SVF: Stromal Vascular Fraction
T2D: Type 2 Diabetes
WHR: Waist-Hip Ratio
WHtR: Waist-to-Height Ratio
A.U.: Arbitrary Units
