# Supplementary material for: CD248 expression in human adipose tissue is upregulated in obesity and following glucocorticoid exposure: a mediator of insulin resistance and adipose dysfunction?
Source: Mol Med. 2026 Oct 7;32:181. doi: 10.1186/s10020-026-01661-8 (PMC13653403; doi:10.1186/s10020-026-01661-8)
Supplement: Supplementary file 1 [file 10020_2026_1661_MOESM2_ESM.docx]

**Supplementary File S2**

**Supplementary File S2 contains additional data corresponding to the analyses presented in the main figures. Supplementary Figure 1** contains total protein expression data corresponding to the analysis presented in the main figures. (**A**) Total protein expression in the primary cohort comparing lean and obese individuals. (**B**) Protein expression stratified by sex in lean and obese individuals from the primary cohort. (**C**) Protein expression comparing subcutaneous (SAT) and omental adipose tissue (OAT) in the surgical cohort. (**D**) Protein expression in adipose tissue following 24-hour dexamethasone treatment. (**E**) Protein expression in isolated adipocytes following 24-hour dexamethasone treatment. **Supplementary Figure 2** shows *CD248* gene expression in a subset of the primary cohort of individuals with no diabetes (ND) and type 2 diabetes (T2D), analysed using transcriptomics data. **Supplementary Figure 3** shows *CD248* mRNA expression in adipose tissue following 24-hour exposure to estrogen (E2). **Supplementary Figure 4** shows *CD248* mRNA expression in human SGBS adipocytes following 2-hour dexamethasone treatment. Data from (23).

**Supplementary Figure 1. Total protein expression corresponding to the analyses presented in the main figures.** (**A**) Total protein expression in the primary cohort comparing lean (L) and obese (O) individuals. (**B**) Protein expression stratified by sex in lean and obese individuals from the primary cohort. (**C**) Protein expression comparing subcutaneous (SAT) and omental adipose tissue (OAT) in the surgical cohort. (**D**) Protein expression in adipose tissue following 24-hour dexamethasone treatment. (**E**) Protein expression in isolated adipocytes following 24-hour dexamethasone treatment.

**Supplementary Figure 2. *CD248* expression in a matched subset of individuals from the primary and and type 2 diabetes (T2D) cohorts.** *CD248* expression was analysed using transcriptomics data in ND (n=12, female: 50%, age: 60 ± 6 years, BMI: 30.1 ± 4.7 kg/m^2^) and T2D (n=12, female: 42%, age: 63 ± 5 years, BMI: 30.4 ± 3.9 kg/m^2^).

**Supplementary Figure 3. CD248 expression in adipose tissue following 24-hour exposure to estrogen.** CD248 mRNA expression in adipose tissue (100 mg) incubated for 24 hours with 1 nM estrogen (E2), normalized to control. n=5.

**Supplementary Figure 4. *CD248* expression in SGBS adipocytes following acute exposure to dexamethasone**. *CD248* mRNA fold change (normalized to control) in SGBS adipocytes treated for 2 hours with dexamethasone (1 μM). Data obtained from (23). Error bars represent lfcSE (DESeq2 log2 fold change standard error), n=3.
